# Choosing in Freedom or Forced to Choose? Introspective Blindness to Psychological Forcing in Stage-Magic

**DOI:** 10.1371/journal.pone.0058254

**Published:** 2013-03-13

**Authors:** Diego E. Shalom, Maximiliano G. de Sousa Serro, Maximiliano Giaconia, Luis M. Martinez, Andres Rieznik, Mariano Sigman

**Affiliations:** 1 Laboratory of Integrative Neuroscience, Physics Department, University of Buenos Aires, Buenos Aires, Argentina; 2 Instituto de Neurociencias de Alicante. Universidad Miguel Hernández - Consejo Superior de Investigaciones Científicas, Sant Joan d'Alacant, Spain; Barrow Neurological Institute, United States of America

## Abstract

We investigated an individual ability to identify whether choices were made freely or forced by external parameters. We capitalized on magical setups where the notion of psychological forcing constitutes a well trodden path. In live stage magic, a magician guessed cards from spectators while inquiring how freely they thought they had made the choice. Our data showed a marked blindness in the introspection of free choice. Spectators assigned comparable ratings when choosing the card that the magician deliberately forced them compared to any other card, even in classical forcing, where the magician literally handles a card to the participant This observation was paralleled by a laboratory experiment where we observed modest changes in subjective reports by factors with drastic effect in choice. Pupil dilatation, which is known to tag slow cognitive events related to memory and attention, constitutes an efficient fingerprint to index subjective and objective aspects of choice.

## Introduction

The word “forcing” is used in stage magic to describe the act in which a subject reports to have made a free decision among equal possibilities while manipulated by the performer whom, then, secretly knows the outcome of the choice. As with other key aspects of the cognitive foundation of magic [1], [2], [3], in psychological forcing the performer uses the fact that only a small and distorted set of information is available to the spectator’s introspective constructs. Physiological forcing is an area of intense debate and study in stage magic [4], [5], [6]. Here we set to capitalize on this basic magical procedure to understand the subjective construct of free or conditioned choice.

As with similar recent enterprises which have linked magic tradition to neuroscience [7], [8], [9], [10] and psychology [11], here we progressively drift from street magic to the laboratory. First, we conducted a one-on-one stage-magic performance, where the magician follows a script designed to inquire participants about their subjective feeling of choosing freely or forced. This questionnaire implemented by the magician was naturally embedded in the performance as part of the show. We assessed two techniques used in stage magic to force a card, namely the Visual Forcing (VF) and the Classical Forcing (CF) [12], [13]. The VF consists of asking one spectator to choose a card by taking a mental picture of it while riffling the whole deck in front of his/her eyes. Since at least 1959 [13] magicians know that subjects tend to choose the last cards of the deck and the ones shown long enough to influence choice, but subtle enough to make the procedure opaque. The CF consists in asking one spectator to pick a card on the deck held by the magician while timing the handling of the deck in such a way that the card to be forced reaches the subject’s fingers at the moment he picks a card. CF has been used by stage magicians for centuries [4].

We then replicated the VF experiment in a laboratory setup where all variables are precisely timed, and measured pupil dilatation as a first step to identify physiological markers of the subjective construct of free or forced choices. We showed a marked blindness in the introspection of free choice in live magical shows: spectators assigned very similar ratings when they chose the card that the magician deliberately enforced them compared to the ratings when selecting any other card. In the laboratory experiment we observed modest changes in subjective reports of free choice by factors which have a drastic effect in choice (for example, card duration and card position in the deck). Finally, pupil dilatation, which was known to tag slow cognitive events related to memory and attention [14], constitutes a rapid and efficient fingerprint to index subjective and objective aspects of choice.

## Materials and Methods

### Participants

All subjects gave written informed consent and were naïve about the aims of the experiments. All the experiments described in this paper were reviews and approved by the ethics committee: “Comité de Ética del Centro de Educación Médica e Investigaciones Clínicas “Norberto Quirno” (CEMIC)” qualified by the Department of Health and Human Services (HHS, USA): IRb00001745 - IORG 0001315.

### Experiment 1

31 subjects completed the task (42% females; mean age 28, range 19–51). Participants were volunteers recruited on a public place. The performer approaches unknown people and asks if they would like to be part of a magic spectacle, mentioning that it is part of an experiment on human decision making. After acceptance, a magician collaborator films and the performer explains that he will first ask the spectator to make some choices and then to rate how free they felt in doing so: if the spectator feels he/she has been somehow manipulated, the rate should be 0; if he/she feels to have made a free decision, without being influenced by external factors, they should rate a 10. They were asked to interpolate their perceived feeling of free choice in a continuous scale between 0 and 10. These results were offline rescaled to the interval 0–1 and inverted (higher values meant feeling more forced), to agree with the second experiment.

The scripted routine was run by a professional magician; he performed a sequence of four different guesses: three chosen cards and then a number freely thought by the spectator. The average duration of the routines was 3∶31 minutes and the results were measured by analysing the filmed videos offline.

### Experiment 2

#### Material

A total of 103 video files were recorded while a trained magician riffled the deck. As in stage performances, the magician forced one card by slightly folding it and hence presenting it for a longer duration. Each video file was tagged frame by frame to time precisely the presentation of each card. On average the riffles lasted 4040 ms (SD 600 ms) and average card exposure time was 82 ms (SD 45 ms). The video files were recorded at 60 frames per second, each frame was presented for 16.6 ms.

#### Procedure

Stimuli were presented on a 19-inch CRT monitor (1024×768 pixels resolution; frame rate 60 Hz). Participants were seated in front of the monitor with the head positioned on a chin rest at a distance of 50 cm from the monitor. Eye movements were recorded with a desktop-mounted, video-based eye tracker (EyeLink 1000, SR Research Ltd., Ontario, Canada) at a sampling rate of 1000 Hz. Nominal average is accuracy 0.5°, and space resolution is 0.01° RMS. Participant's gaze was calibrated with a standard 13-point grid for both eyes. All recordings and calibration were binocular. Only right eye data was used for the analysis. The experiment was implemented in Matlab (Mathworks, Natick, MA) using Psychophysics toolbox (Brainard, 1997).

#### Participants

20 subjects completed the task (40% females; mean age 27, range 20–61). Participants were volunteers recruited from the general population of the University of Buenos Aires and were paid for their participation. All subjects were native speakers of Spanish who reported normal (or corrected to normal) vision.

#### Analysis

The visual forcing was considered effective when the participant chose either one of the two last cards (forced by position), or one of the two longest cards in each riffle (forced by duration). ROC curves were calculated for each individual, considering separately both forcing by duration and forcing by position. The procedure to calculate ROC curves is the following. for each threshold value across the interval [0,1], two quantities are calculated: the proportion of high-SRF in forced trials p(high SRF | forced) (the number of SRF values greater or equal to the threshold in forced trials, divided by the number of forced trials), and the proportion of high-SRF in non-forced trials p(high SRF | non-forced) (the number of SRF values greater than the threshold in non-forced trials, divided by the number of non-forced trials). ROC area of 0.5 (a straight line) corresponds to individuals with poor introspection.

For the statistical analysis of pupil size, subject’s averages were calculated for each condition. With these curves, a 2-way ANOVA (objective forcing and subjective report) was performed at running bins of 50 ms. A result was considered significant when at least 5 consecutive bins were below p = 0.05.

## Results

### Experiment 1

We measured psychological forcing in one-on-one stage performances (see Video S1. People in the video have given written informed consent, as outlined in the PLOS consent form, to publication of the video.). A professional magician ran a scripted routine where he performed a sequence of four different guesses: three chosen cards and then a number freely thought by the spectator. The first two guesses followed respectively Visual Forcing and Classic Forcing procedures. If the forcing was unsuccessful the magician pulled out the chosen card by subtle prestidigitation using various techniques, including an ordered deck, false shuffling, and a palming technique consisting in hiding a card in the magician’s hand. In the third guess the spectator was asked to think and say aloud any card of the deck which then the magician managed to appear as the only face down card in a deck of face up cards. In the fourth guess, the spectator was asked to choose a number which then “magically” appeared written in a piece of paper held by the magician. Here, prestidigitation was used to write the number in the paper after it was chosen. None of the spectators participating in this study detected the magician prestidigitation in any of the four guesses.

After each guess, independently of whether the guessed card had been forced or not, the spectator was asked whether he felt that he had chosen the card freely or, instead, if he/she thought that the magician had biased his/her selection. Participants were asked to report this in a scale from zero to one. This questionnaire did not disrupt the performance; instead it flew coherently as part of the spectacle. A simple inspection of the videos showed that all participants were vividly engaged throughout the performance.

Visual and classical psychological forcing was effective (participant chose the card that the magician attempted to force) respectively for 14 (45%) and 17 (54%) out of 31 participants. Both forcing procedures were simultaneously effective for 8 (26%) which is the closest integer to the expected value (7.66) of simultaneous forcing probability, given that the likelihood to be forced by each procedure is independent. Although these percentages of forcing success could seem low, they are in fact relatively high given the context of the experiments. Thus, and contrary to what could be expected, experienced magicians rarely rely on the probability of forcing success alone. They often make use of parallel lines of action to cover potential forcing failures, including the introduction of alternative endings, such as in our routine when, as stated above, the magician pulled out the chosen card anyway by subtle prestidigitation. Moreover, when performing street magic, magicians try not to use these physiological forcing routines right at the beginning of their show; but rather they usually do it after conditioning the spectator in different ways to increase success rates. One such strategy is, for example, to allow the mark to take a card freely before introducing the classical forcing. In addition, magicians are trained to be quite good at identifying the more susceptible subjects in their audience to be forced. In our case, none of these precautions were taken, our subjects were randomly recruited in a public place (a shopping mall) and the experiments began right away, to avoid introducing bias and/or other confounding factors.

Subjective reports of forcing (SRF) were very similar and did not differ significantly for both forcing procedures: 0.132±0.037 for visual forcing and 0.113±0.039 (SE) for classical forcing (paired t-test: t(30) = 0.39, p = 0.69) (Table 1). More importantly, we did not observe any difference when splitting these reports between effective and ineffective forcing procedures (unpaired t-test: t(60) = 0.60, p = 0.55, mean SRF effective forcing: 0.138±0.043, mean SRF not forced: 0.106±0.032 SE). We then submitted the data to an ANOVA with the number of guess as main factor (the third and fourth guesses were not driven by forcing procedures). The effect of guess number (1 to 4) on SRF was not significant (2-way ANOVA: effect of guess number: f(3,86) = 0.91, p = 0.44, effect of participant: f(30,86) = 2.03, p = 0.006). Together, these results indicate that the subjective perception of being forced did not change when subjects were actually being forced (successfully or not) or whether the choice they made relied on a mechanical action (a card riffle) or the generation of an internal thought out of a number of options (choosing a number).

**Table 1:** Subjective report of forcing in experiment 1.

	VF	CF	Card guess	Number guess
Ineffective forcing	0.118 (0.033)	0.093 (0.059)	0.093 (0.031)	0.050 (0.023)
Effective forcing	0.150 (0.072)	0.129 (0.053)		

Mean (SE) values of subjective report of forcing (SRF) in the Experiment 1, a scripted routine of one-on-one magician performance. The values are in a scale from 0 to 1, being 0 feeling free and 1 being forced.

### Experiment 2

To examine these findings in quantitative detail we conducted a simplified version of this experiment in a laboratory setup. We filmed 103 different card riffles produced by a magician. As in stage performances, the magician forced one card by slightly folding it and hence presenting it for a longer duration. We then analyzed the videos offline, frame by frame, to time the duration of each card. Participants were asked to fixate in the centre of the deck and their gaze was controlled.

**Figure 1:**
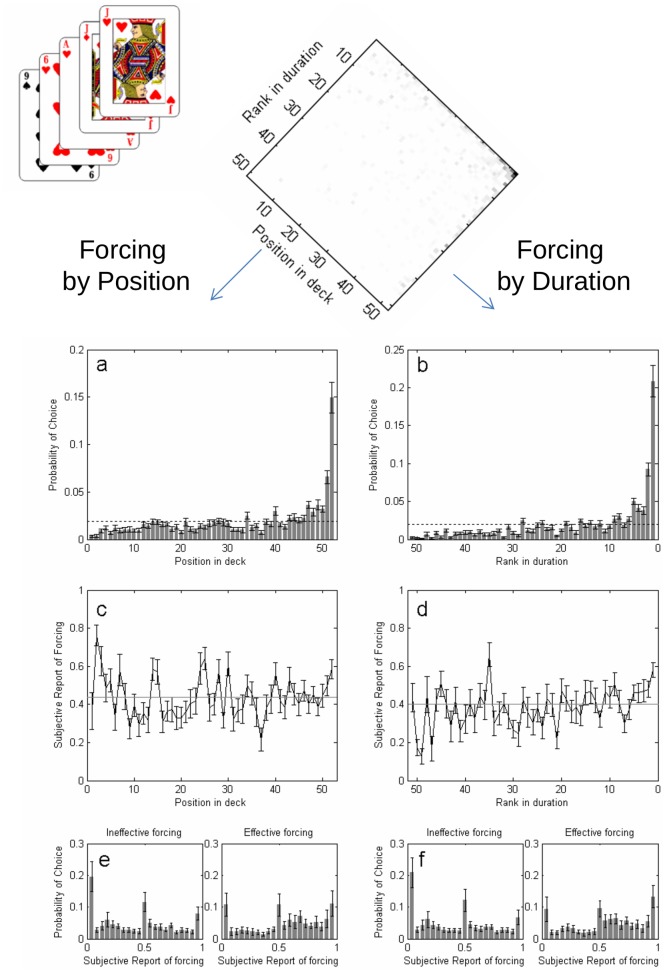
Forcing by position and forcing by duration. Top panel: Probability of choice as a function of both position and rank in duration. a, b- Probability of choice as a function of position in deck and rank in duration. Dashed lines correspond to chance level. c, d- Subjective report of free choice as a function of position in deck and rank in duration. Dashed lines correspond to global mean. e, f- Distribution of SRFC for effective and ineffective forcing by position and duration. Errorbars are standard errors of subjects’ means.

We analyzed choice based on two regressors which are well known to affect selection as documented both in psychological research [15], [16] and in the magic literature [12]: duration and position in the deck. This analysis revealed indeed a sharp and very significant effect of both factors in choice (Figure 1). The card presented for longer duration (number 1 in rank of duration) was chosen in 20.8% (SE 2.1%) of all trials and the last card of the deck was selected in 15.0% (SE 1.6%) of all trials. Both values are very large and highly significant (p<0.00001) compared to chance levels (1/49 or about 2%, dashed line, which corresponds to the average, 49.2 (SD 2.3) cards presented in each riffle, which is slightly less than the total number of cards -52).

In contrast with this sharp transition in likelihood of choice, subjective report of forcing showed a weak (but significant) dependence on these two forcing parameters (Figure 1 c-d). SRF showed a significant difference in the mean value for both forcing conditions, when comparing forced choices (two longest or two last cards) to unforced choices (paired t-test of mean subject’s values; forcing by position: t(19) = 3.88, p = 0.001; forcing by duration: t(19) = 5.42, p = 0.00003). An analysis of the full distribution of SRF showed a shift to higher values when participants chose the last card in the deck or the one presented for longer duration compared to other cards. However, in both cases the distribution reflects many instances in which participants chose the forcing card yet reporting free choice, or conversely, many instances in which participants did not choose any of the forcing cards and yet reported that they perceived that their choice was forced. A direct comparison of the SRF in the laboratory and the live one-on-one situation shows significantly higher values in the later (unpaired t-test: t(49) = 7.44, p<0.00001; mean SRF in lab setup: 0.46±0.17; mean SRF in one-on-one live situation: 0.13±0.20 SD) suggesting that subjects tend to trust more their own actions in a normal human interaction and further stressing the significant bias that could ballast cognitive studies in artificial, laboratory set-ups.

**Figure 2:**
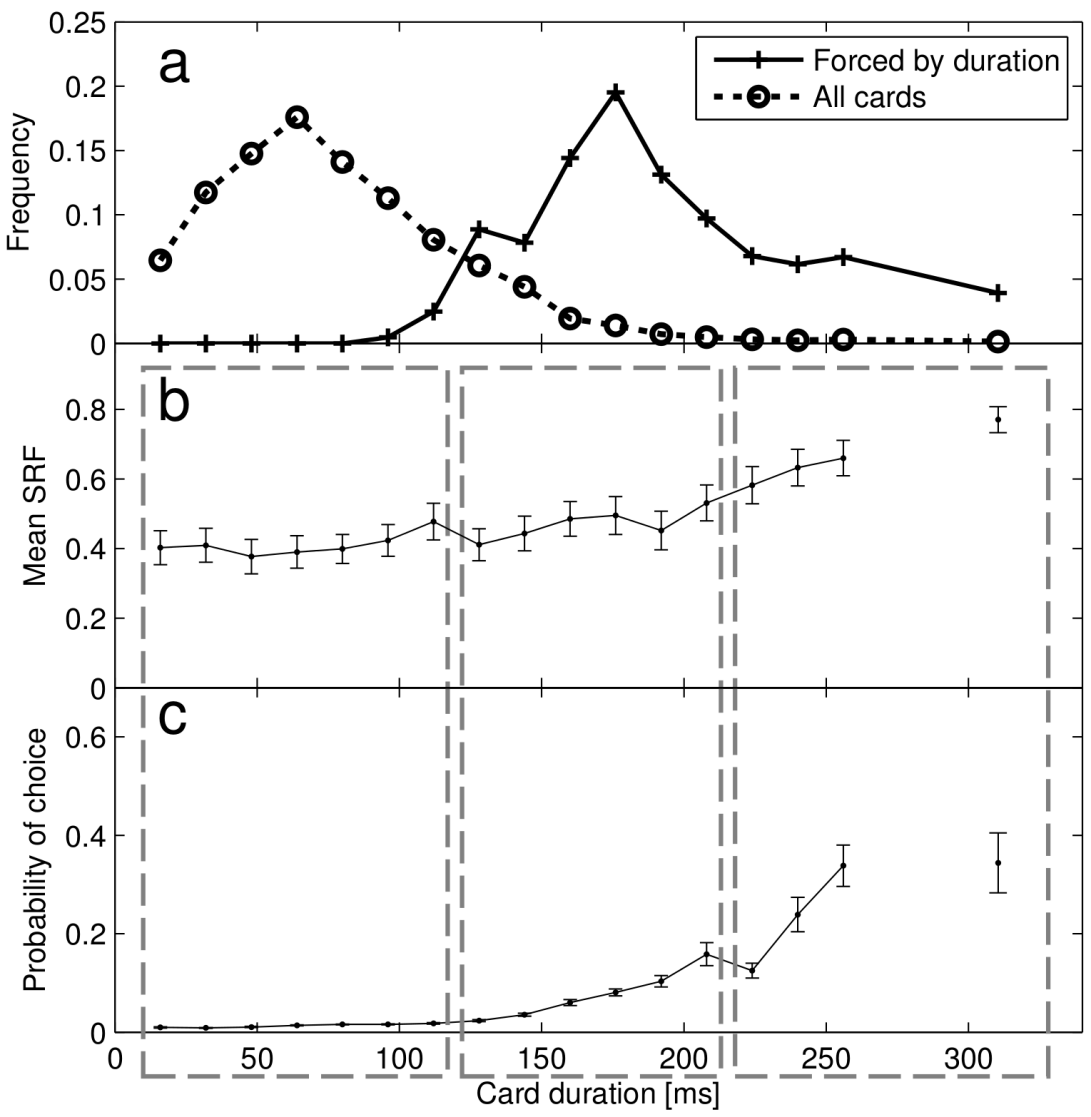
Probability of choice and SRF vs card duration. a- Histogram of card duration for all the cards, and the cards considered forced by duration (the longest two cards of each riffle). b- Mean subjective report of forcing as a function of chosen card duration. c- Probability of choosing cards as a function of card duration. Cards presented for more than 256 ms were combined in a single datapoint. Errorbars are standard errors of subjects’ means.

Since the distribution of durations varied slightly in each riffle, for robustness the analysis described above was based on the rank of duration and not on absolute duration. Next, to specifically address the relevant time-scales, we analyzed choice and SRF as a function of card duration (Figure 2). This analysis showed three distinctive regimes. Fluctuations in durations below 120 ms did not affect choice (Figure 2c) or subjective reports (Figure 2b). Fluctuations in duration beyond 200 ms conditioned choice, yielding very high values of forcing with high probabilities, but this was accompanied with an explicit report by the subjects that they felt the chosen card had been forced. We identified an intermediate regime – broadly confined between 120 and 200 ms - which of course is of most interest to magic (Figure 2a)- where fluctuations in duration significantly change the likelihood of a card being chosen (an almost 10-fold increase building up to 10% probability) without affecting the subjective perception of free choice.

**Figure 3:**
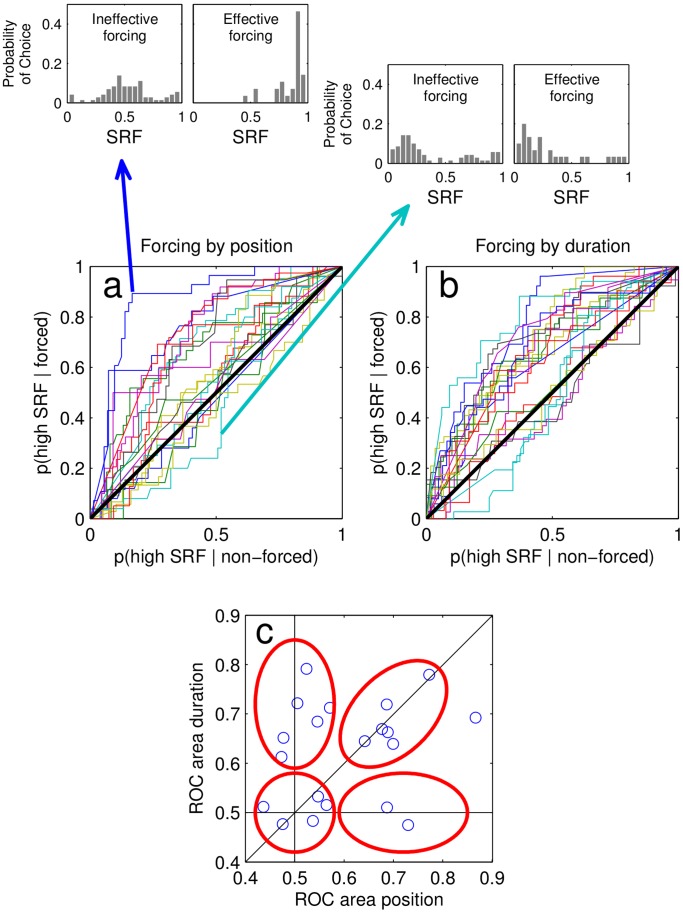
ROC curves of each participant. ROC curves of each participant, for a- forcing by position, and b- forcing by duration. Insets: SRF distributions of forced and non-forced choices, for the highest- and lowest- ROC area subjects. c- Individual ROC area by duration as a function of ROC area by position.

The results from the two experiments coherently show systematic opaqueness in the capacity of a subject to identify whether temporal properties of the stimuli play a role conditioning their choice. This can be explicitly measured by a Receiver Operator Characteristics (ROC) analysis [17] which, in a non-parametric way serves to estimate how much the SRF is a faithful estimator of whether the card had been actually forced. This accuracy is often referred in the psychological literature as Type-II performance, to distinguish it from Type-I performance [18], [19] which reflects accuracy in an objective task. ROC analysis can then assign to each participant whether the SRF reports are good descriptors of objective forcing. A participant with accurate Type-II performance will typically show high values of SRF when the card was forced and low values of SRF when choosing a card which was not forced. In comparison a participant with inaccurate Type-II performance (low ROC values) will produce very similar distributions of SRF for objectively forced and not-forced choices (Figure 3 top insets).

The distribution of ROC curves varied widely across subjects (Figure 3a–b) (mean ROC area forced by position: 0.61±0.12 SD; forced by duration: 0.62±0.10 SD). Interestingly, for both categories, a substantial fraction of participants showed ROC areas close to 0.5 (main diagonal line), indicating complete blindness to perceive psychological forcing.

A subject by subject analysis of Type-II performance based on position and duration (Figure 3c) revealed the following pattern: a) 5 subjects with almost chance Type-II performance, b) 6 Participants showed accurate Type-II performance based on duration, but chance performance based on position, c) 2 subjects with accurate Type-II performance based on position, but chance performance based on duration, d) 7 participants showed relatively accurate values of Type-II performance based on both variables, i.e. could identify forced choices based on the two more canonical forcing parameters. While the sample is not sufficiently large to make quantitative arguments on whether these groups form significant clusters, both ROC distributions look bimodal (Figure 3a–b) and the scatter plot reflects a broad variability among subjects which can be reasonably arranged in the classes described above. This result shows that - as is well known to magicians - some subjects are highly vulnerable to magic since they are unable to detect external objective forcing.

The dissociation which we observed between objective measures of choice and subjective reports of perceived free choice raises the question of whether physiological markers can distinctively index each process. To make a first step in this direction we recorded the pupil dilatation locked to the chosen card. We analyzed the dynamics of pupil size distinctively according to subjective (perceived forced or perceived free) and objective (chose the forced card or another in the deck) factors. To avoid confounds based on choices of the last cards of the deck (when there is an abrupt change in the visual display) we only considered forcing by duration and considered only chosen cards which were not in the last tercile of the deck.

**Figure 4:**
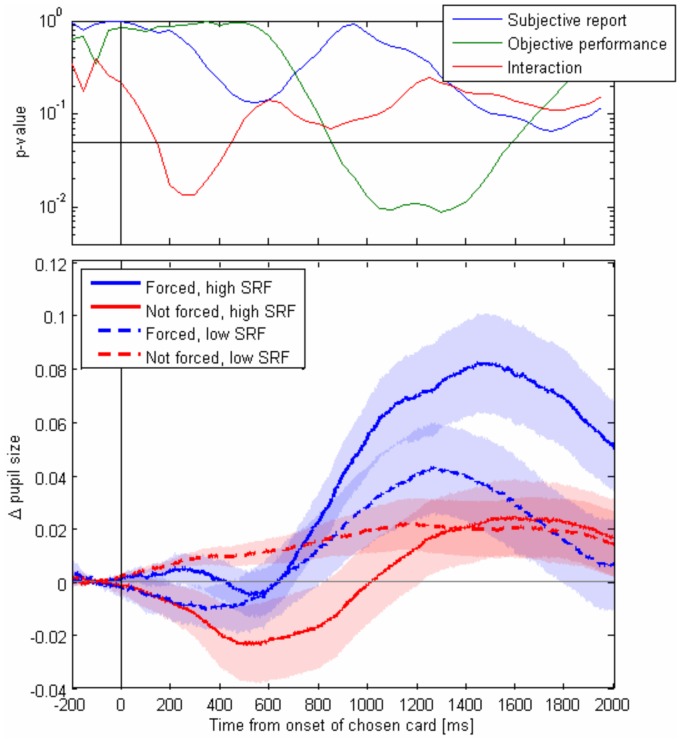
Pupil dynamics. Lower panel- Baseline-corrected pupil size locked to the appearance of the chosen card, for all conditions of forcing and subjective report of forcing. Higher panel- p-value significance resulted from 2-way ANOVA performed at running bins of 50 ms. Horizontal line corresponds to p = 0.05. Errorbars are standard errors of subjects’ means.

Pupil size showed a rich dynamics (figure 4 lower panel), revealing a relatively late peak (∼1300 ms), strongly modulated by objective factors (i.e. whether the chosen card was the one presented for longer duration in the riffle). Instead, early dynamics of pupil dilatation showed a dip which was more prominent when the participant chose a card which was forced but without perceiving it as forced and a very small build-up of pupil-size when the subject chose a card which was not forced and not perceived as forced. These observations are quantified by an ANOVA analysis performed at running bins of 50 ms, which showed an early (200–300 ms) interaction between subjective (perceived as forced or not) and objective (forced or not) factors and a late (1000–1400 ms) main effect of the objective factor (figure 4 upper panel).

## Discussion

Freely choosing a card in a riffle was heavily weighted by the position of the card, with the majority of subjects opting for one of the last two cards of the deck. This is a well known fact to psychological research since the early investigations of Hermann Ebbinghaus [20] As expected we observed a strong recency effect (a tendency to recall the last items of a list) but not a primacy effect (better recall for the first items of the list), since the later decreases for longer lists, especially when items are presented quickly [15], two features exaggerated in the riffles used here. Similarly, fluctuations in duration in RSVP are expected to affect the saliency of an item in the list [16].

Hence the factors governing choice observed in this experiment are a mere corroboration of well known principles in psychology. These principles are also very familiar to the magic literature and tradition [4], [13]. The novelty of our work is to investigate how these factors affect the subjective perception held by the participant on whether the choice he made was “free” or not. Our results showed a marked introspective blindness, an inability of subjects to understand when a choice they had made had been forced by these main factors. This results sum to other demonstrations of introspective blindness in decision making where factors which govern decisions and choices are not accessible to meta-cognitive judgments such as confidence [21], [22], [23], [24], [25] or temporal properties of the elements constitutive of the decision [26], [27]. Similarly, our results are also in-line with several studies demonstrating that individuals often confabulate about the rationale to explicitly justify their choices [28], [29], [30], [31], [32].

The SRF introduced here can be thought of as the inverse of the subjective sense of agency (SSA). A central question in this field is whether our conscious experience of agency (SSA) is confabulatory, as suggested by Nisbett and Wilson [28], Wegner [32], and Johanson et al [30] or the result of processing genuine information about the actions and their consequences, as proposed by Moore and Haggard [33]. Moore and Haggard [33] defend that our SSA is almost never confabulatory but relays on feedforward neural mechanisms that are part of the same motor programs that control our self-initiated actions [34]. In support of this claim, Fried et al. [35] have shown that minimal electrical stimulation of the supplementary motor area of neurosurgical patients triggered an urge to perform a particular movement even in the absence of the corresponding motor behaviour. On the other hand, Moore and Haggard [33] point out that our reasons for action could be much more susceptible to retrospective influences and confabulation. Therefore, the interaction between the experience of an action and the conscious thinking about the reasons for that action could confound the conclusions of previous reports. Our results contribute to this argument by showing that SRFs remain the same in the different experimental conditions, suggesting that subjects confabulate their own SSA, particularly in situations in which they think they chose freely a card that was physically forced to them.

The analysis of individual ROC curves reflected a broad variability. This indicates that the blindness to identify forcing mechanisms in decision making varies widely across different individuals. Similarly, meta-cognitive ability (i.e. to typically assign high confidence in correct trials and low-confidence scores to incorrect trials) in perceptual decision making varies across subjects [21], [22]; a variance which can be partly accounted by gray matter volume in the anterior prefrontal cortex [21] and which can be intervened by temporal inactivation of the prefrontal cortex with TMS [24]. Whether meta-cognitive blindness to different elements of the decision (quality of the decision relating to confidence, origins of the decision relating to free-will and free-choice, constituents of the decision) involve a shared system or functions should be a matter for future investigation.

Finally, we investigated the dynamics of pupil size as a proxy of the neural correlates of SRF during the magic trick. Pupil size is considered an effective measure of mental effort, attention and cognitive control [14]. Moreover, pupil size is thought to index activity of the locus coeruleus. The locus coeruleus (LC) is the most important norepinephrine (NE) nucleus in the brain, with bursts of activity following behaviourally relevant sensory events [36]. Hence LC activity, and its covariate measured as pupil-size is a good marker to indicate saliency of an item in an RVSP and candidate markers for subjective variables (assigning to an internal or external driven choice) tagged to this item. Our observation that in fact pupil size dynamics can index objective (longer duration of an item) and subjective (perceived as being forced or not) has three important implications: 1) First it builds up on two recent studies showing that pupil dynamics, in spite of its intrinsic slow response function, can be used to tag fast transients in attention and cognitives state [25], [37]. 2) It indicates, indirectly, that fluctuations in neuromodulators which regulate pupil-size such as NE co-occurring with stimulus presentation can modulate the subjective construct of a choice and 3) it shows a rich dynamics with different phases revealing non-trivial interactions between objective (forced or not) and subjective (perceived as forced or not) at different stages of the pupillary response.

Although attempts to establish a relationship between magic and psychology are not new [11], [38], [39], recently there has been a renewed interest in using magic techniques as a vehicle to investigate more systematically the human brain and behaviour [1], [2], [9], [40]. These studies have addressed several aspects of perception including eye-movements [41], [42], [43], [44] attention [7], [45], [46], [47], visual system limits [8], self-deception [29], [30], and brain-processing of causal effects [48]. Our study follows this fruitful tradition, by using magic to address the construction of free will.

A basic working hypothesis in stage magic is that this introspective construction is not a binary function but instead a weighted combination of several factors which can be worked out in construction to maximize the illusory perception of free choice. Hence, the magic literature on physiological forcing is full of techniques designed to increase an individual’s perception of having made a free decision while being forced. Our approach was inspired by this well established fact by magicians, showing a concrete example of the deep insight that stage performers can bring to the scientific studies of the human mind.

## Supporting Information

Video S1 **Example video of Experiment 1.** A professional magician ran a scripted routine performing four different guesses: three chosen cards and then a number freely thought by the spectator. The first two guesses followed respectively Visual Forcing and Classic Forcing procedures.
(AVI)
